# How to build an effective research network: lessons from two decades of the GARNet plant science community

**DOI:** 10.1093/jxb/eraa397

**Published:** 2020-09-08

**Authors:** Geraint Parry, Yoselin Benitez-Alfonso, Daniel J Gibbs, Murray Grant, Andrea Harper, C Jill Harrison, Eirini Kaiserli, Sabina Leonelli, Sean May, Sarah McKim, Steven Spoel, Colin Turnbull, Renier A L van der Hoorn, James Murray

**Affiliations:** 1 GARNet, School of Biosciences, Cardiff University, Cardiff, UK; 2 Centre for Plant Sciences, University of Leeds, UK; 3 School of Biosciences, University of Birmingham, UK; 4 School of Life Sciences, University of Warwick, UK; 5 Department of Biology, University of York, UK; 6 School of Biological Sciences, University of Bristol, Bristol, UK; 7 Institute of Molecular, Cell and Systems Biology, University of Glasgow, UK; 8 Exeter Centre for the Study of the Life Sciences, University of Exeter, UK; 9 Nottingham Arabidopsis Stock Centre, School of Biosciences, University of Nottingham, UK; 10 School of Life Sciences, University of Dundee and James Hutton Institute, UK; 11 Institute of Molecular Plant Sciences, University of Edinburgh, UK; 12 Department of Life Sciences, Imperial College, UK; 13 Department of Plant Sciences, University of Oxford, UK; 14 MPI of Molecular Plant Physiology, Germany

**Keywords:** Collaboration, community, GARNet, integration, network, research, resources, training

## Abstract

Successful collaborative research is dependent on excellent ideas and innovative experimental approaches, as well as the provision of appropriate support networks. Collaboration requires venues, infrastructures, training facilities, and, perhaps most importantly, a sustained commitment to work together as a community. These activities do not occur without significant effort, yet can be facilitated and overseen by the leadership of a research network that has a clearly defined role to help build resources for their community. Over the past 20 years, this is a role that the UKRI-BBSRC-funded GARNet network has played in the support of the UK curiosity-driven, discovery-led plant science research community. This article reviews the lessons learnt by GARNet in the hope that they can inform the practical implementation of current and future research networks.

## Teaser

Lessons learnt from the activities of the GARNet research network in their support of the global plant science community are discussed.

## The origin and development of the Genomic Arabidopsis Resource Network (GARNet)

The *Arabidopsis thaliana* genome was published in December 2000, bringing plant science research into the genomics era and strengthening the foothold of this unassuming weed as the primary plant model organism (Arabidopsis Genome Initiative, 2000). To take advantage of the rapidly increasing set of molecular tools that were emerging for use with Arabidopsis, Ottoline Leyser, then based at the University of York, led a successful bid to the BBSRC Investigating Gene Function (IGF) initiative to establish the Genomic Arabidopsis Resource Network (GARNet). GARNet was to provide reliable, user-driven, and publicly available functional genomic resources for Arabidopsis researchers. The 4 year project was led from York, coordinated by Ottoline and Karin Van de Sande, whilst the activities were overseen by an advisory group of academics.

During this first phase of funding, GARNet facilitated the set-up of transcriptomic, bioinformatic (both at Nottingham), proteomic (Cambridge), and metabolomic (Rothamsted) facilities as well as provision for the generation of mutant populations and clones at the John Innes Centre. In addition, GARNet hosted a successful annual meeting, the first of which was attended by 250 delegates in 2000. Since its inception, GARNet has had close links with the Nottingham Arabidopsis Stock Centre (NASC). This facility was established in 1990 and helped place the UK as a global leader within the Arabidopsis research community. GARNet has played an advisory role in the ongoing evolution of NASC activities, and NASC Director Sean May has contributed to the GARNet advisory committee since its inception.

After four productive years of facilitating the adoption of functional genomics, the role of GARNet was revised to support the emergence of systems biology as a tool for network analysis and gene discovery. From 2004 to 2009, the Arabidopsis and wider plant community obtained over £31 million investment in plant systems biology throughout the UK. During this period, GARNet activities were led from the University of Edinburgh by Andrew Millar as principal investigator (PI) and Ruth Bastow as the full-time coordinator.

The foresight to be involved with emerging technologies such as systems biology was again demonstrated as GARNet was funded by BBSRC for another 5 years (2010–2014), with Jim Beynon at the University of Warwick as PI and Ruth Bastow, Irene Lavagi, Charis Cook, and Lisa Martin as the coordination team. On this occasion, the proposal explored the use of synthetic biology in plant science, helped expand the uptake of systems biology approaches, promoted translational research, and supported the international community through administration of the Multinational Arabidopsis Steering Committee (MASC). The success of this funding also allowed GARNet to play a major role in shaping the wider UK plant science community as they led in the formation of the UK Plant Science Federation.

The linkages between GARNet and the UK plant science community were expanded during a fourth round of BBSRC funding between 2015 and 2020. In this period, GARNet promoted the use of new technologies, many developed and validated in Arabidopsis that were relevant for all plant scientists to facilitate the translation of ideas from models to crops. These activities had particular focuses on the emerging field of gene editing and on establishing the software and hardware infrastructures needed to deal with big data. This final UKRI-BBSRC-funded iteration of GARNet activities was led by Jim Murray at Cardiff University with Geraint Parry as the full-time coordinator, with support from Ruth Bastow and Lisa Martin.

From July 2020, the GARNet leadership team was unable to obtain further UKRI funding to continue activities. However, the GARNet advisory committee maintains that UK plant science requires a community-facing network that can integrate researchers through broad knowledge exchange, highlighting of and training provision in new technologies that reach between experimental systems. In general, the GARNet advisory committee proposes that a key role for a future research network is to encourage the interaction between researchers and extant research infrastructures, such as multiomic or microscopy facilities, and ensure that previous capital investments are maximized and supported by world class expertise.

One challenge in supporting research networks is to define the mechanism through which funding is provided for their activities. Since 2004, GARNet was funded through responsive mode funding calls, in which it directly competed with conventional research proposals. Hence, although a successful network will add value to a wide set of researchers, the network in essence drew funding away from the very researchers it aims to support. Hopefully the successes of the GARNet network will be a motivation for funders to develop schemes through which successful community-enhancing networks can obtain longer term support.

## The changing landscape of UK plant science

**Fig. 1.**
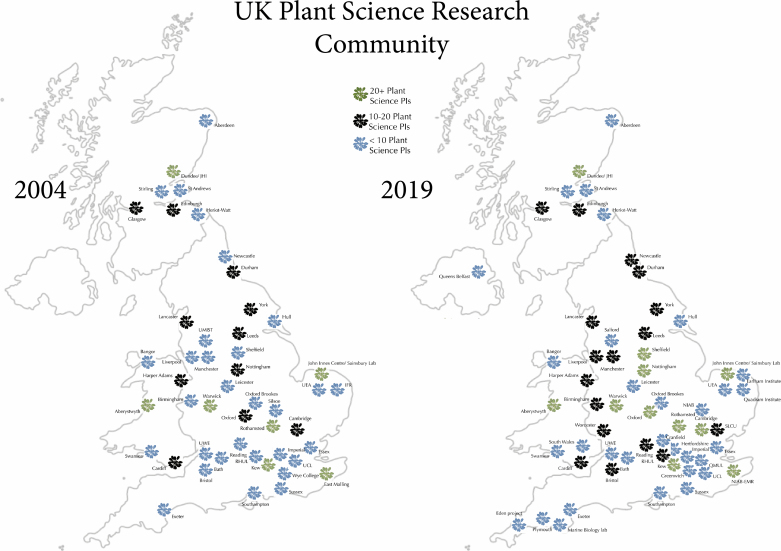
UK Plant Science in 2004 and 2019. Data for 2004 were taken from Edition 1 of the GARNish newsletter (https://garnetcommunity.org.uk/sites/default/files/newsltr/garnish_july04.pdf) and may exclude PIs from departments that focus on more applied research. Data for 2019 were taken from prior knowledge and online searches. At the very least, this suggests that there has not been a decline in the number of PIs whose primary research focus is in some area of plant science.

The past two decades have brought a radical transformation in the ways in which plant science research is conducted, including the increasingly pivotal role of data science skills, the integration of molecular analysis with developmental, evolutionary, and environmental insights, and the translational advances in applied crop science. Over the same period, many, but not all, exclusively botanical- or plant science-focused degree programmes have disappeared from UK higher education (Drea, 2011). Nevertheless, it appears that the UK plant science research community has at the very least remained at a similar size if not expanded. Figure 1 shows a comparison between 2004 and 2019 in the number of plant science-focused researchers at UK universities and research institutes.

Collaborative plant science projects have also benefited from the emergence of formal regional research networks. These include the N8 Agrifood network and the Great Western 4 (GW4) consortium which have been successful in bringing together researchers to pool expertise and expand access to research infrastructures. These relationships have succeeded in supporting shared infrastructures or obtaining funding for graduate training networks. These achievements are greater than might be possible by individual institutions acting alone.

GARNet has been active throughout this time, with an overarching remit to add value to the investments made in the UK plant science community, both from UKRI and from other sources. This was initially achieved by providing access to facilities for emerging omic technologies and then through promotion and training in the new technologies needed for the UK plant community to be internationally competitive. The UK remains a world leader in plant science research, as measured by both citation rate and H-index of publications (Scimagojr, 2020). Much of the research in these publications has been underpinned by access to resources initially funded by GARNet as well as through its many training activities. Over the past 5 years alone, this has included organization of 14 conferences and training events.

The management structure that links the GARNet leadership team and its advisory committee to the wider community has allowed its activities to stay ahead of the technology curve and take a leading role in the training of plant scientists in cutting-edge research techniques.

Many of the lessons learnt from 20 years of GARNet activities are discussed below (Box 1). We hope that they will provide valuable advice for those planning to establish new initiatives or expand existing networks.

**Box 1. Advice for community-focused research networks**

1. Initially incentivize community participation
2. Stay ahead of the technology curve
3. Build an advisory group who are invested in the network
4. Add value by securing additional funding to support community-facing activities
5. Integrate with the wider community
6. Engage the next generation
7. Employ a Project Manager

### Initially incentivize community participation

Network projects usually arise from the motivation of a small group of like-minded individuals. However, over the longer term, a network will only succeed if it has more extensive buy-in from the wider community. The wheels of this process can be greased by providing financial or access-driven incentives to draw people into the network. If there is research or technology development funding associated with the network then this will motivate academics and others to become involved with the process.

GARNet initially benefitted from the BBSRC IGF programme that provided direct funding to help set up omic resources that had an Arabidopsis focus. This attracted those academics in the position to lead these community facilities. A similar strategy is now employed in 2020 by both the PhenomUK program (PhenomUK, 2020), which is funded through the UKRI-BBSRC Technology Touching Life scheme, and multiple ‘Networks in Industrial Biotechnology and Bioenergy’ (NIBB) schemes (UKRI-BBSRC, 2020). These offer up to £50 000 in Proof of Concept funding for either technology development in plant phenotyping or for development of academic–industrial interactions, respectively. Despite this funding being relatively small, it is clearly welcomed as an available route to support research projects. This type of interaction will hopefully ensure that these networks can expand to become part of the wider research landscape.

In GARNet’s early years, the community was also incentivized through the scheduling of an annual meeting that brought together UK-based Arabidopsis researchers and international experts. Financial support from GARNet allowed these meetings to be inexpensive and developed a sense of community, particularly for PhD students and postdocs, which encouraged delegates to participate in other GARNet activities. Establishing an annual meeting meant that each year could focus on a novel technology or ongoing area of research in order to keep the community informed about new developments. Although this began as an Arabidopsis-focused event, it soon evolved to promote technologies that were relevant to plant scientists irrespective of their organism of research. It is clear from this example that hosting a regular gathering of network members further engages the community and helps establish its activities and shape priorities.

### Stay ahead of the technology curve

The 20 year success of GARNet was built around the incentivized awareness, engagement, and understanding of technology. Any research network must be continuously forward looking, assessing new technologies and techniques as they emerge. This was facilitated by the ability of the GARNet advisory group to predict the importance of new technologies and by GARNet’s capability to respond to community training needs. Specifically, the initial GARNet proposal was funded in response to the imminent release of the Arabidopsis genome sequence, whilst subsequent GARNet proposals coincided with the emergence of systems and/or synthetic biology approaches as important parts of the research portfolio. An important caveat to this advice is to not lose sight of the reason for the technology, which ultimately should be used to support scientific inquiry, as highlighted in 2004 by then GARNet chairman Ian Furner in the December 2004 edition of the GARNish newsletter; ‘GARNet has been very much about technology but the real point of all the technology is to find out fun stuff about biology’ (GARNish Newsletter, 2004).

The advice for future networks is to ensure that their leadership team has the foresight to plan to provide the wider community with something they might not even know they need. From a GARNet perspective, this was exemplified in 2013 by promotion of ‘Opportunities in Plant Synthetic Biology’ that included introducing novel genome assembly techniques and heterologous expression systems (Cook *et al.*, 2014).

It is of course not possible to predict all approaching advances in technology, especially if a grant period extends for 5 years. Therefore, where possible, it is important that the funding model has the agility to respond to emerging technologies by allocating funds to areas not directly earmarked in the successful proposal. This ability to respond to emerging challenges was highlighted during the GARNet2020 grant period. The initial proposal was submitted in early 2014, at which time the potential of CRISPR (clustered regularly interspaced palindromic repeats)-mediated gene editing had not fully emerged. However, through the allocation of funding for unspecified future events, the details of which would be decided by the GARNet advisory committee, it allowed the flexibility to organize events that focused on gene editing technologies that reach across the entire plant science community (Parry *et al.*, 2016; Parry and Jose, 2018; Parry and Harrison, 2019). Therefore, important advice for any new network is to ensure that during the funding period there is reasonable flexibility to enable it to respond to unexpected technological developments.

Despite the clear importance to provide training in new technologies, it is critical that there is simultaneous support for the infrastructure that underpins the outputs of these technologies. Over the past 20 years, the uptake of new technologies has been characterized by an enormous increase in the generation of big data. This has driven the necessity for the integration of experimental outputs with the digital infrastructure. Therefore, any new research network should be involved with both ends of the data journey, from training in the techniques to generate the data and in the infrastructure to manage and allow reuse of the data.

### Build an advisory group who are invested in the network

Academics are busy people and are pulled in many directions. However, the success of any academic-focused research network will rely upon the recruitment of members who are committed to contribute to its activities. These network advocates greatly enhance the chance of the network succeeding. Conversely, it is important that the network leadership team does not place onerous demands on an advisory committee to prevent membership becoming a chore.

From the second GARNet grant period in 2004, the advisory committee comprised at least nine members who were elected for a period of 3 years. The location of GARNet advisory committee meetings moved around the UK so as to even out the burden of travel time. An annual rotation of three members ensured that new ideas were regularly introduced to the committee. The wider UK plant science community was engaged with this process as the ‘GARNet electoral register’ is comprised of the entirety of UK-based plant science academics (currently >600 members).

A fresh impetus of new ideas is achieved not only by the annual rotation of committee membership but also by an excellent gender balance, variety of experience levels, and geographic distribution, which ensures that a mix of voices can input their advice. This was achieved through a two-step election process in which UK-based plant science PIs nominated and then voted for their peers who would become part of the GARNet advisory committee. As such, we hope that any unconscious biases from the advisory committee were removed by having the community make decisions on their behalf. GARNet made it clear that any eligible PI was welcome to join the committee and, over recent years, this was reflected by election of PIs whose primary research does not use Arabidopsis, thus expanding the areas of expertise of the advisory committee. Table 1 shows that 37% of the 51 academics who have sat on the GARNet advisory committee are female. Although this is not perfect, we feel that over 20 years it represents a commitment to equality. These PIs are distributed throughout the UK, increasing the likelihood that resulting GARNet activities are shared around the country. Future networks should look to replicate this wide geographic distribution to ensure they operate as a true national network.

**Table 1.** Members of the GARNet Advisory Committee (2000–2020)

Name	Institution	Starting year on GARNet Advisory Committee														
John Doonan	Aberstywyth University									2012						
Jim Murray	Cardiff University								2011							
Colin Turnbull	Imperial College														2017	
Robert Sablowski	John Innes Centre						2009									
Saskia Hogenhout													2015			
Smita Kurup	Rothamsted Research								2011							
Alessandra Devoto	Royal Holloway University of London						2009									
Jonathan Jones	Sainsbury Lab, Norwich		2005													
Cyril Zipfel										2012						
David Salt	University of Aberdeen										2013					
Julia Coates	University of Birmingham							2010								
Daniel Gibbs														2016		
Claire Grierson	University of Bristol		2005													
Antony Dodd											2013					
Jill Harrison														2016		
Ian Furner	University of Cambridge	2000														
Paul Dupree				2006												
Alex Webb							2009									
Ian Henderson												2014				
Phil White	University of Dundee, James Hutton Institute				2007											
Claire Halpin						2008										
Sarah McKim															2017	
Keith Lindsey	University of Durham	2000														
Patrick Hussey						2008										
Heather Knight										2012						
Andrew Millar	University of Edinburgh	2000														
Steven Spoel													2015			
Christine Raines	University of Essex												2015			
Sabina Leonelli	University of Exeter						2009									
Nick Smirnoff								2010								
Anna Amtmann	University of Glasgow					2008										
Eirini Kaiserli																2018
Phil Gilmartin	University of Leeds	2000														
Brendan Davis				2006												
Stefan Kepinski							2009									
Yoselin Benitez-Alfonso																2018
Anthony Hall	University of Liverpool									2012						
Simon Turner	University of Manchester	2000														
Sean May	University of Nottingham	2000														
Zoe Wilson				2006												
Malcolm Bennett									2011							
Zoe Wilson												2014				
Nick Harberd	University of Oxford	2000														
Miltos Tsiantis					2007											
Ian Moore								2010								
Nick Harberd											2013					
Renier Van Der Hoorn																2018
Julie Gray	University of Sheffield		2005													
Jim Beynon	University of Warwick				2007											
Katherine Denby												2014				
Murray Grant														2016		
Ottoline Leyser	University of York	2000														
Andrea Harper															2017	
Murray Grant	Wye College	2000														

GARNet’s commitment to the future of UK plant science is confirmed by the regular involvement of early career faculty members on the advisory committee. Since 2014, eight of the 15 elected members have been within the first 8 years in an academic position (Henderson, Spoel, Gibbs, Harrison, Harper, McKim, Kaiserli, and Benitez-Alfonso). Academics who participate on the GARNet advisory committee will naturally have a community outlook and so GARNet has been able to expand its wider network through the external interactions of advisory committee members. Over recent years, these have included organizational roles with the Society of Experimental Biology (SEB), the British Society of Plant Pathology, and the UK Plant Science Federation.

Providing a clear benefit to advisory committee members for their contribution will ensure that they remain invested in promoting the network. Over the past few years the GARNet coordinator worked with committee members to organize symposia in their areas of research interest including ‘Integrating Large Data into Plant Science: from Big Data to Discovery’ (Leonelli *et al.*, 2017), ‘Natural Variation as a Tool for Gene Discovery and Crop Improvement’ (Henderson and Salt, 2017), and ‘From Proteome to Phenotype: Role of Post-translational Modifications’ (Spoel, 2018).

GARNet took the additional step to ensure that it was fully connected to its community by inviting a representative from its funder (UKRI-BBSRC) to sit on the advisory committee. This is not so that the funder would influence GARNet activities but rather that they were made aware of matters arising from within the community. Over the past decade, this relationship has included extremely useful discussions regarding the funding landscape, for which the BBSRC shared non-public information that GARNet could then report back to the community. In general, this collaborative relationship between a grant recipient and its funder(s) provides a two-way dialogue that can benefit both parties. This transparency is further demonstrated by the online publication of the minutes from each advisory committee meeting. This allows the community to be fully aware of network activities and provides an open conduit between the community and network.

### Add value by securing additional funding to support community-facing activities

For a network to be successful, it must both integrate into the existing community and use its resources to benefit that community. Even the most community-minded PI has limited time to be involved with external training events and conferences. Therefore, a network should look to add value for its community by gaining support from outside organizations that might have resources to fund relevant activities. This may involve applying for conference funding from learned societies, arranging travel grants for early career researchers, bringing together academics to apply for collaborative grants, or leading journal-supported community initiatives. For example, the SEB has Animal, Cell, and Plant Sections for which it organizes meeting and workshops. GARNet has a longstanding relationship with the SEB and over the past 5 years this has resulted in GARNet obtaining over £50 000 in support from the SEB for meetings and training events. This enabled GARNet to direct both SEB funding and the work of the SEB directorate toward providing events specifically for the plant science community it represents. This takes the burden away from academics and others to commit their time to being involved with these type of funding applications.

GARNet has used its community connections to be involved in a range of collaborative grants aimed at building UK research infrastructure. In this regard, the network coordinator can heavily input toward time-consuming writing of proposals. This was a successful strategy during the third grant period in which GARNet was involved with the CyVerseUK/iPlant project that aimed to federate hardware and software resources under the US CyVerse infrastructure (UK Research and Innovation, 2020). This project linked the Earlham Institute with the Universities of Nottingham, Liverpool, and Warwick. Furthermore, GARNet members contributed to the COPO (COpenPlantOmics) project that facilitated the management of complex plant science datasets and linked the Earlham Institute, EMBL-EBI, and the Universities of Oxford, Warwick, and York (COPO Project, 2020).

### Integrate with the wider community

Although GARNet began with the remit to provide funding for resources that focused on Arabidopsis research, over the second half of its lifetime the activities of the leadership team, advisory committee, and coordinator were integrated with a wider group of UK and global plant scientists. This included a BBSRC-funded administrative role with the Multinational Arabidopsis Steering Committee (2020), a leadership role with the INDEPTH Cost Action (INDEPTH, 2020), and knowledge exchange relationships with other UK community research networks. This strategy demonstrates that a network should have value that extends beyond its initial objectives so as to demonstrate to funders that their investment is reaching a wider community than originally planned.

In 2010, the leadership team at GARNet identified that the UK plant science community lacked a single unified voice so, in collaboration with the leaders of Brassica, Monogram, and Solanaceae networks, came together to establish a ‘Federation of UK Plant Science Communities’ that was supported by a GARNet-managed website (Leonelli *et al.*, 2012). The permanent staff supported by GARNet funding managed these resources for the benefit of the wider community. Early successes of this network included winning funding from the SEB, the Gatsby Charitable Foundation, the Biochemical Society, and the British Society of Plant Pathology to support a national ‘UK PlantSci meeting’ and produce a widely circulated report on ‘Current Status and Future Challenges of the UK plant Science Community’ (Royal Society of Biology, 2020a).

Over past 5 years, the now renamed UK Plant Science Federation (UKPSF) has evolved to be a special interest group of the Royal Society of Biology and, with extensive input from the GARNet Advisory Committee, recently produced the ‘Growing the Future’ report (Royal Society of Biology, 2020b) on the future of UK plant science. UKPSF activities are now managed by the Royal Society of Biology and, as it does not rely on grant funding to maintain its activities, it will hopefully ensure UKPSF’s longevity.

Any nascent network can benefit from widening its influence and using the strength of its funding to support other communities. This will benefit a network over the longer term via the connections it can make throughout the community.

As well as developing connections within its research area, any network will benefit by expanding to establish relationships with multidisciplinary groups. GARNet worked with CPIB at the University of Nottingham to initiate the Mathematics and Plant Science Study Groups. These events invited a group of mathematicians to tackle previously ‘unmodelled’ problems in plant science. These events lasted 7 years and directly resulted in (at least) two peer-reviewed publications (Antoniou-Kourounioti *et al.*, 2012; Nelson *et al.*, 2012; GARNet, 2020).

GARNet also interacted with the history and philosophy of science research community through the invitation to Sabina Leonelli to join the advisory committee as an *ex-offico* member. Leonelli investigates the policy implications and technical mechanisms that allow the effective management of big data so that they are findable and reusable. This provided GARNet with an expert perspective and clear voice within discussions on the future of mechanisms to promote data management, sharing, and reuse (Bastow and Leonelli, 2010; Leonelli *et al*., 2013, 2017). The focus on data usage and availability was an important part of the successful proposal that secured the fourth round of GARNet funding in 2014.

Engaging a multidisciplinary group of collaborators during advisory committee meetings and event organization demonstrated that GARNet had a willingness to embrace new ideas and interactions. Any new network would benefit from looking at the options to expand its circle of interactions. This strategy requires the leadership team and advisory committee to have the necessary vision to move network activities into unexpected areas, even if they may at times be outside the advisory committee’s ‘comfort zone’.

### Engage the next generation

The success of any network relies on engaging not only with established PIs but also with the next generation of scientists. GARNet’s knowledge exchanges activities catered to early career researchers by offering affordable opportunities to attend and participate in conferences, provide information about available job and research opportunities, interact with other organizations to facilitate funding for overseas conference travel, and, most importantly, provide training in new technologies. Each of these activities aims to build a general appreciation of research network activities and that these younger scientists will continue to see this value as their careers progress.

Community engagement now relies not only on routine E-mail correspondences or occasional newsletters but also on dedicated use of multimedia outputs and social media for the circulation of new findings, events, and opportunities. These multi-media interactions are surprisingly time-consuming so it is important that sufficient time is given in order to maximize their impact. Finally, it is important to ensure that a network chooses a social media profile that is closely connected to its activities so that it is easy to find. This will work better than the use of a more enigmatic ID such as @GARNetweets! Despite this, the @GARNetweets twitter account gains ~30 followers a week with a high engagement rate of 1.7%.

### Employ a project manager

Despite the best intentions of the leaders of any network, its effective management will probably be more time-consuming than they might imagine. Therefore, irrespective of the size of the network, it is important that a project manager is employed to direct its everyday activities. This might be a full-time or part-time role but is critical to liaise with the advisory committee, apply for external funding, organize events, deal with grant administration, and disseminate outputs. GARNet has historically employed an experienced coordinator who becomes an active member of the advisory committee, yet a new network might choose a less integrated manager. Overall, a project manager adds value both by overseeing the network and also by allowing the academic leadership to focus their own research programmes, which after all has brought them to the position to take the lead in a community research network.

## Conclusion: GARNet perspectives

The landscape of UK plant science has changed significantly over the past two decades. The revolution in plant genomics was led by Arabidopsis researchers but now has been followed by the establishment of equivalent tools that will allow discovery-led research in crop plants with more complex genomes (Adamski *et al.*, 2020). Nevertheless, models such as Arabidopsis remain the key test beds for developing new techniques and tools, and translating emerging technologies from outside the plant field that can subsequently be utilized by the wider plant science community.

UKRI-BBSRC remains the primary individual grant funder of world-leading UK plant science research. Each year since 2014, UKRI-BBSRC has funded an average of 38 responsive mode grants that have some focus on plant science; for an approximate value of £19 million. This compares with an approximate value of €7 million per year awarded through European Research Council funding for individual plant science projects. In addition, UKRI provides institutional support for plant science research at the John Innes Centre, IBERS, The Earlham Institute, the Quadram Institute, and Rothamsted Research. Over this time period, the Gatsby Foundation has provided significant support for The Sainsbury laboratories in Norwich and Cambridge as well as for numerous PhD studentships.

It is impossible to fully assess the change in the number of UK-based PIs over the last 15 years who conduct plant science research, but Fig. 1 suggests that at the very least this number has remained stable. However, it is likely that there has been a shift from discovery-led research to more applied research. Indeed, over the past 5 years, the number of grant proposals that plan to use Arabidopsis to answer fundamental questions in plant science has declined and often been replaced by proposals across research areas that will use a variety of other plant species, including crops, trees, and newer model organisms such as *Nicotiana benthamiana*. An additional and unfortunate consequence of broadening the funded portfolio of UK plant science is that the overall network remains fragmented between researchers involved in ‘fundamental’ or ‘applied’ research. This increases the need for flexible research networks that can bring together researchers who work in different areas.

Through its two decades, GARNet has demonstrated that adaptability is necessary for longevity. It has adapted its activities to move from supporting the use of Arabidopsis-focused research infrastructures, through promotion of new research areas such as systems and synthetic biology, through to the more recent focus on providing training opportunities in new technologies. With the support of the GARNet advisory committee, the GARNet PIs have gained BBSRC-responsive mode funding in preference to more conventional research proposals. Hopefully, the lessons learnt from 20 years of community involvement can make GARNet an exemplar for future research networks.

## Data Availability

The data on the number of UK-based plant science principal investigators (Fig. 1) are available from Geraint Parry upon request.
